# Carbon fixation by basalt-hosted microbial communities

**DOI:** 10.3389/fmicb.2015.00904

**Published:** 2015-09-07

**Authors:** Beth N. Orcutt, Jason B. Sylvan, Daniel R. Rogers, Jennifer Delaney, Raymond W. Lee, Peter R. Girguis

**Affiliations:** ^1^Bigelow Laboratory for Ocean SciencesEast Boothbay, ME, USA; ^2^University of Southern CaliforniaLos Angeles, CA, USA; ^3^Biological Laboratories, Harvard UniversityCambridge, MA, USA; ^4^Stonehill CollegeNorth Easton, MA, USA; ^5^School of Biological Sciences, Washington State UniversityPullman, WA, USA

**Keywords:** geomicrobiology, biogeochemistry, microbial ecology, ocean crust, basalt, deep biosphere, carbon fixation

## Abstract

Oceanic crust is a massive potential habitat for microbial life on Earth, yet our understanding of this ecosystem is limited due to difficulty in access. In particular, measurements of rates of microbial activity are sparse. We used stable carbon isotope incubations of crustal samples, coupled with functional gene analyses, to examine the potential for carbon fixation on oceanic crust. Both seafloor-exposed and subseafloor basalts were recovered from different mid-ocean ridge and hot spot environments (i.e., the Juan de Fuca Ridge, the Mid-Atlantic Ridge, and the Loihi Seamount) and incubated with ^13^C-labeled bicarbonate. Seafloor-exposed basalts revealed incorporation of ^13^C-label into organic matter over time, though the degree of incorporation was heterogeneous. The incorporation of ^13^C into biomass was inconclusive in subseafloor basalts. Translating these measurements into potential rates of carbon fixation indicated that 0.1–10 nmol C g^-1^_rock_ d^-1^ could be fixed by seafloor-exposed rocks. When scaled to the global production of oceanic crust, this suggests carbon fixation rates of 10^9^–10^12^ g C year^-1^, which matches earlier predictions based on thermodynamic calculations. Functional gene analyses indicate that the Calvin cycle is likely the dominant biochemical mechanism for carbon fixation in basalt-hosted biofilms, although the reductive acetyl-CoA pathway and reverse TCA cycle likely play some role in net carbon fixation. These results provide empirical evidence for autotrophy in oceanic crust, suggesting that basalt-hosted autotrophy could be a significant contributor of organic matter in this remote and vast environment.

## Introduction

Oceanic crust is the largest aquifer on Earth, with the entire volume of the ocean circulating through the crust on the order of every 10^5^–10^6^ years (Fisher and Becker, 2000). This “subsurface ocean” within the oceanic crust is a site of geologically rapid chemical exchange between the crust and the oceans, which has ramifications on global chemical cycles (Edmond and Von Damm, 1983; Stevens and McKinley, 1995; Alt and Mata, 2000; Bach and Edwards, 2003; Bach et al., 2004). Moreover, this aquifer comprises a massive potential habitat for microbial life, yet very little is known about the rates of microbial activity in this environment (Orcutt et al., 2011b). Knowledge of metabolic reactions occurring in the oceanic crust is sparse because accessing pristine samples for microbiological investigation from this environment is technologically challenging. Most information about microbial activity in ocean crust comes from (i) the quantity and speciation of chemical constituents within rocks or fluids collected from the subsurface or at the seafloor (e.g., Bach and Edwards, 2003; Wheat et al., 2003; Rouxel et al., 2008; Wheat et al., 2010), (ii) functional genes observed in environmental DNA (Mason et al., 2010), or (iii) incubations of mineral substrates in the environment or in the laboratory as a proxy for natural processes (Edwards et al., 2003a,b; Templeton et al., 2009; Toner et al., 2009; Orcutt et al., 2011a). Two recent studies provided the first empirical assessment of microbial sulfate reduction activity in hot and anoxic subsurface ocean crust settings (Lever et al., 2013; Robador et al., 2014).

Determination of rates of microbial activity is essential for evaluating the impact of crustal biosphere microbial activity on global elemental budgets and for understanding the mechanisms for growth and survival of life in this environment. In particular, the sources, sinks, and cycles of carbon in the crustal biosphere are important to understand, since (i) carbon is the primary building block for life, (ii) igneous oceanic crust is a sink for carbon from the oceans (Alt and Teagle, 1999), and (iii) the crustal reservoir is immense (Orcutt et al., 2011b). The potential impact of microbial carbon fixation in the crustal biosphere is unknown, however, as empirical assessments of rates of autotrophy are lacking. One recent study suggests that organic matter in hot and anoxic ocean crust is chemosynthetic in origin (McCarthy et al., 2011), while another recent study suggests that this system is net heterotrophic (Lin, 2013); however, rates of carbon cycling are unconstrained. Are there significant rates of endogenous carbon fixation that alter the inorganic carbon budget in the crust, therefore impacting the sink of inorganic carbon in vein formation? Or, conversely, are the rates of autotrophic carbon production overshadowed by heterotrophic consumption of carbon?

Using theoretical modeling, Bach and Edwards (2003) predicted that aerobic and anaerobic (NO_3_-dependent) Fe- and S-oxidation could support ∼50 × 10^10^ g C year^-1^ of autotrophic primary production in oceanic crust, with H_2_-based sulfate reduction and methanogenesis supporting an additional 1–10 × 10^10^ g C year^-1^ (assuming production of hydrogen from water-rock reactions). Furthermore, these calculations suggest that H_2_ reduction of Fe oxides, nitrate, and oxygen could also support 10^10^–10^12^ g C year^-1^ each, assuming available hydrogen and oxidized species are present. These autotrophic primary production rates of >10^12^ g cellular carbon per year are similar in magnitude to the rate of heterotrophic organic consumption in sediment, indicating that autotrophic processes in the oceanic crust could be significant contributors to the local, and potentially global, C cycle.

A survey of environmental DNA from seafloor-exposed basalt collected from the Juan de Fuca Ridge revealed the presence of genes for proteins involved in carbon fixation processes (RuBisCO, ATP citrate lyase, and methyl-coenzyme M reductase; Mason et al., 2009), providing further support for the existence of autotrophy in this environment. Other recent work demonstrates significant extracellular enzyme activity for organic substrates from seafloor-exposed basalt-hosted microbial communities, with activities that are comparable to other marine environments such as marine sediment, marine snow particles, and mangroves (Jacobsen Meyers et al., 2014). However, the methodology in that study could not distinguish between autotrophic and heterotrophic activity, leaving relative rates of autotrophic activities on basalt unknown. Thus, to our knowledge, there are no empirical measurements of carbon fixation activity from either seafloor-exposed or subsurface ocean basalts.

**FIGURE 1:**
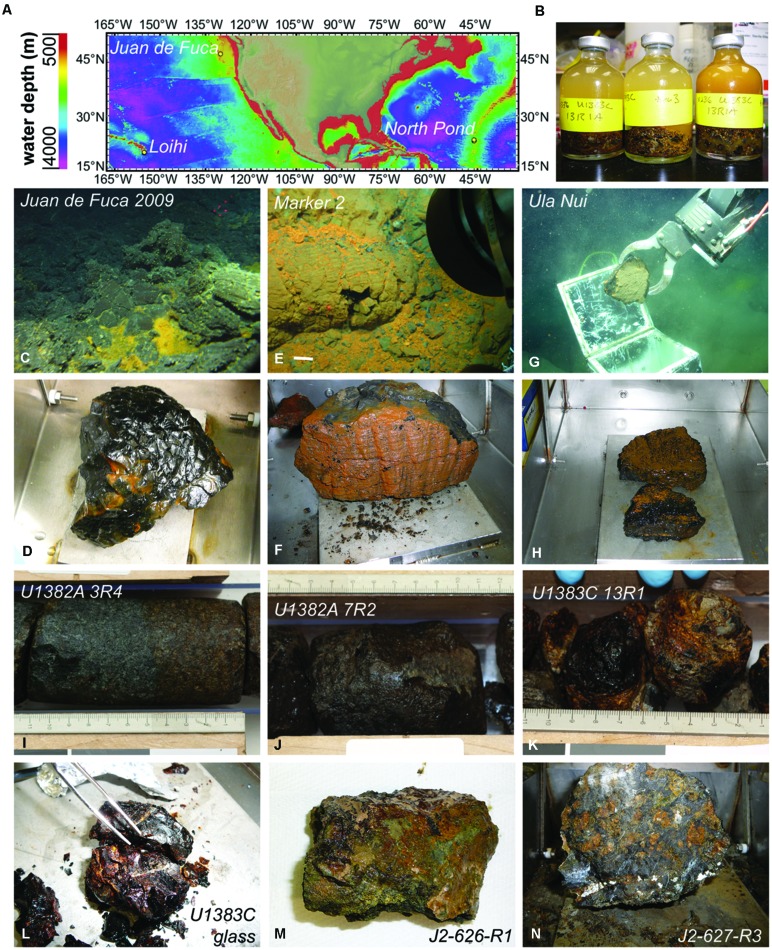
**Locations and photographs of samples used in the study. (A)** Location map with bathymetry contours created using GeoMapApp (Ryan et al., 2009). **(B)** Example of sample incubation set up. **(C)** Location of glassy basalt (in **D**) collected from the Juan de Fuca Endeavor Ridge. **(E)** Iron oxide stained basalts (in **F**) collected from within Pele’s Pit on the summit of the Loihi Seamount offshore Hawai’i. **(G)** Collection of iron oxide stained basalt (in **H**) from the Ula Nui ultradiffuse hydrothermal vent site at the base of the Loihi Seamount. Massive basalts with pervasive oxidation were collected from Hole U1382A sections 3R **(I)** and 7R **(J)** at North Pond, whereas glassy basalts were collected from Hole U1383C sections 13R **(K)** and 19-21R **(L)**. Manganese encrusted altered basalts were collected from the seafloor of North Pond on dives J2-626 **(M)** and J2-627 **(N)**. Photos in **(B,D,F,H,L–N)** by B. Orcutt; photos in **(C,E,G)** are copyright Woods Hole Oceanographic Institution; photos in **(I–L)** previously published (Expedition 336 Scientists, 2012c,d) and reprinted here with permission of the Integrated Ocean Drilling Program.

To constrain the potential for chemoautotrophic carbon fixation by basalt-hosted microbial communities, and to identify microbial groups involved in autotrophy, we conducted incubation experiments with stable isotope tracers, as well as quantitative analysis of target gene abundance, with seafloor basalt samples collected from a mid-ocean ridge seamount (the Axial Seamount on the Juan de Fuca Ridge, NE Pacific Ocean), a mid-plate hotspot (Loihi Seamount, offshore Hawaii), and on the flank of a mid-ocean ridge (North Pond, Mid-Atlantic Ridge, Atlantic Ocean; **Figure 1**). Subsurface basalt samples from North Pond collected during scientific ocean drilling were also included in the experiments, to examine the potential for this process in the crustal deep biosphere. Basalt samples with a range of alteration from these different locations were incubated with ^13^C-labeled sodium bicarbonate over the course of hours to days to months, and incorporation of ^13^C into organic matter was evaluated using isotope ratio mass spectrometry (IRMS) as a proxy for carbon fixation activity. Our results reveal the potential for autotrophic carbon fixation in basalt biofilms, as demonstrated by the enrichment of ^13^C-label of organic matter over time. We compare these potential rates of carbon fixation to recently available data on extracellular enzyme activity of basalt-hosted microbial communities to surmise the relative contribution of autotrophic processes to fueling the basalt biosphere. When one considers the relatively large volume of this habitat, even the modest carbon fixation rates determined in this study suggest that basalt-hosted autotrophy may be a significant source of fixed carbon for the deep ocean environment.

## Materials and Methods

### Sample Collections and Descriptions

Nine different basalt samples were used in this study, collected from three different crustal formation areas (**Figure 1A**; **Table 1**). One glassy, seafloor-exposed basalt came from the ASHES vent field in the Axial Seamount volcano caldera on the Juan de Fuca Ridge off the western coast of North America (**Figure 1C**). The sample was collected in 2009 with the *Alvin* submersible during dive AD-4527 on RV *Atlantis* cruise AT15-51 (Sample JdF2009). The basalt piece had a thick (up to 1 cm depth) glassy rim overlying moderately to sparsely vesicular cryptocrystalline groundmass (**Figure 1D**). A film of iron oxide discoloration was evident in the contact between the glass and groundmass. Two altered, seafloor-exposed basalts were collected from the Loihi Seamount off the coast of the big island of Hawai’i in October 2009 by *ROV Jason-II* during the FeMO2009 cruise on the R/V *Kilo Moana* (cruise KM09-23): one from “Marker 2” in Pele’s Pit on the summit of the Loihi Seamount (**Figure 1E**, Sample Marker 2), and one from the Ula Nui vent field at the base of the Loihi Seamount (**Figure 1G**, Sample Ula Nui). Sample Ula Nui, collected on dive J2-477, was glassy, highly vesicular, and friable, contained ∼3 mm olivine phenocrysts, and displayed visible iron oxide staining (**Figure 1H**). Sample Marker 2, collected on dive J2-481 from an area away from diffuse venting, was pillow basalt with an altered rind (**Figure 1F**). Both of the Loihi basalts used in this study were also used in a recent study to examine extracellular enzymatic activity by basalt-hosted microbial communities (Jacobsen Meyers et al., 2014). The remaining six basalts were collected from “North Pond,” a small sediment pond located to the west of the Mid-Atlantic Ridge (**Figure 1A**). Four subsurface samples were collected in October 2011 during scientific ocean drilling on Integrated Ocean Drilling Program (IODP) Expedition 336 from Hole U1382A Sections 3R4 (**Figure 1I**) and 7R2 (**Figure 1J**), and from Hole U1383C Section 13R1 (**Figure 1K**) and a mixture of altered glassy basalt from sections 19R-21R (**Figure 1L**). Samples U1382A 3R4 and U1382A 7R2 were phyric massive basalts with varying degrees of oxidation, while the samples from U1383C were extrusive glassy basalts with abundant iron oxide staining (Expedition 336 Scientists, 2012c,d). Finally, two seafloor-exposed rocks were collected from outcrops surrounding North Pond in April 2012 by ROV *Jason-II* on dives J2-626 (**Figure 1M**) and J2-627 (**Figure 1N**) during the MSM20-5 cruise on the R/V *Maria S. Merian*. The J2-626-R1 rock was a breccia of mm- to cm-sized clasts of aphanitic basalt in a greenish-gray matrix, while the J2-627-R3 rock was a highly serpentinized harzburgite (25–30% orthopyroxene surrounded by completely serpentinized olivine and with chrysotile-filled veins; W. Bach, personal communication).

**Table 1:** Characteristics of the basalt samples used in this study, including date of collection, cruise and dive number of collection, water depth of sample, depth below seafloor, latitude, longitude, and weight percent organic carbon (OC; listed as average ± 1 standard deviation; *n* = 3–12).

Sample ID	Date	Cruise/dive	Water depth [m]	Depth [mbsf]	Lat.	Lon.	OC [%]
JdF 2009	2009-08-22	AT15-51/AD4527	1545	0	45.934	-130.014	0.030 ± 0.007
Ula Nui	2009-10-07	KM09-23/J2-477	4987	0	18.707	-155.184	0.058 ± 0.022
Marker 2	2009-10-12	KM09-23/J2-481	1178	0	18.909	-155.257	0.058 ± 0.024
U1382A 3R4	2011-10-06	IODP X336/NA	4483	118	22.589	-46.082	0.045 ± 0.024
U1382A 7R2	2011-10-06	IODP X336/NA	4483	154	22.589	-46.082	0.023 ± 0.017
U1383C 13R1	2011-10-25	IODP X336/NA	4414	173	22.802	-46.053	0.018 ± 0.001
U1383C glass	2011-10-25	IODP X336/NA	4414	∼220	22.802	-46.053	0.025 ± 0.016
J2-626-R1	2012-04-23	MSM20-5/J2-626	3949	0	22.469	-46.036	0.053 ± 0.036
J2-627-R3	2012-04-24	MSM20-5/J2-627	3875	0	22.751	-46.056	0.024 ± 0.006

*AD, HOV Alvin dive; AT, R/V Atlantis; J2, ROV Jason II dive; KM, R/V Kilo Moana; IODP, Integrated Ocean Drilling Program; mbsf, meters below seafloor; MSM, R/V Maria S. Merian; NA, not applicable.*

### Incubations

Upon retrieval of the samples on board the research vessel, all seafloor-exposed rocks and surrounding water from the plastic sampling containers (“bioboxes”) were immediately transferred to glass jars and placed at 4°C until processing, as described elsewhere (Santelli et al., 2008). Basalts were then transferred to ethanol- and flame-sterilized steel processing trays and subsampled with ethanol- and flame-sterilized chisels and tweezers. The experiments utilized the glassy rims of the basalts, which were removed, broken into smaller pieces (<1 cm diameter) and transferred to sterile glass serum vials (30–100 ml volume, depending on experiment) containing 0.2-mm-mesh filter-sterilized oxic bottom seawater. All samples were maintained at 4°C.

The subsurface basalts from North Pond were collected by extended core barrel (XCB) coring as described in detail elsewhere (Expedition 336 Scientists, 2012a,b). Briefly, samples were transferred from the core barrel into sterile plastic bags using 500°C heat-sterilized aluminum foil. Samples were rinsed three times with 0.2-μm-mesh filter-sterilized, autoclaved seawater to remove potential exterior contamination (Expedition 336 Scientists, 2012c,d), as XCB cores are often contaminated on recovery (Smith et al., 2000); this may have also removed some surface-exposed biofilm microbes. Contamination testing with fluorescent microspheres indicated a lack of contamination of all rocks after washing, with the exception of some of the U1383C glassy basalts, as described in detail elsewhere (Expedition 336 Scientists, 2012c,d). Samples were broken apart in a flame-sterilized, stainless steel processing tray as above, then crushed into smaller fragments in a flame-sterilized mechanical metal rock mill.

Basalt fragments (5–20 cm^3^) were transferred to sterile and baked glass serum vials (to remove organics, vials had been heated to 500°C for 2 h), which were filled to overflowing with sterile oxic seawater then sealed with autoclaved butyl rubber septa and aluminum crimp seals. Multiple replicate bottles were prepared for each sample to enable as many time series as possible with the limited sample volume, including a no-tracer-addition control.

Time series samples were injected with a small volume of 0.2-μm filter-sterilized ^13^C-bicarbonate-labeled solution (in sterile filtered seawater) to achieve the following starting concentrations: JdF2009 incubations received a final concentration of 0.75 mM ^13^C-labeled bicarbonate against a background of seawater bicarbonate (∼2 mM, or, 27% ^13^C label); the Ula Nui and Marker 2 rock incubations received a final concentration of 2.7 mM ^13^C-labeled bicarbonate in background bottom seawater (57% ^13^C label); the subsurface North Pond basalt incubations contained a final concentration of 1 mM ^13^C-labeled bicarbonate in background surface seawater (33% ^13^C label); and the seafloor-exposed North Pond basalt incubations contained a final concentration of 4.5 mM ^13^C-labeled bicarbonate in surface seawater (69% ^13^C label). Vials were incubated in the dark at 4°C until sampling. At each time point, the vials were opened and rock fragments were transferred to sterile plastic centrifuge tubes and frozen for shore-based DNA and organic carbon extraction and analysis. For the JdF2009, Marker 2, and Ula Nui samples, time series were stopped after 1 h, 1 day, and 1 week of incubation. The North Pond samples were incubated for 2-weeks, 2-months, and 4-months intervals. Final concentrations of dissolved inorganic carbon were not measured, as rates of carbon consumption were presumed to be significantly slow compared to the bulk pool size, which is supported by our results.

### Organic Carbon Quantification, Carbon Stable Isotopic Analysis, and Potential Fixation Rates

The carbon content and stable carbon isotopic composition of biofilms on the incubated basalts were determined by IRMS analysis of subsamples of the basalts that had been stored frozen. Aliquots of the basalt were bathed in acidified deionized water (pH 3, with HCl) in acid-cleaned glassware, with addition of fresh solution repeated after 1 h, to remove any traces of carbonate. Remaining rock residue was dried under vacuum at 60–70°C, then pulverized in an acid-cleaned mortar and pestle to a sand-like granularity. Aliquots were transferred to acid-washed glass vials or aluminum foil prior to analysis. Dried samples of basalt (20–70 mg) were placed into tin foil capsules for isotopic analysis of ^13^C/^12^C ratios. Samples were analyzed using a Costech elemental analyzer in line with a Micromass Isoprime continuous flow stable isotope mass spectrometer. The zero-blank autosampler was purged with helium gas to reduce background atmospheric contamination. Sample size was optimized to gain a sample peak equivalent or higher to reference material with 0.08 mg of carbon used to calibrate sample runs. Results are presented in the standard δ notation, where isotopic ratios (R) are expressed in per mil (‰) differences relative to the conventional standard, the PeeDee Belemnite limestone where (Eq. 1):

$$\delta =\left(\frac{R_{sample}}{R_{s\,\tan dard}}-1\right)\times 1000$$

The isotopic ratio (^13^C/^12^C) of the standard reference material (*R_standard_*) was 0.0111796, and routine precision for δ^13^C was 0.3‰ based on the standard deviation of ten replicate measurements of reference material.

Potential carbon fixation rates were determined based on ^13^C-bicarbonate label uptake into organic matter over time. First, the isotopic ratio of the sample (*R_sample_*) was calculated by rearranging Eq. 1. The mass change in carbon labeling between time points and the time zero sample was calculated based on the change in isotopic ratios (Δ ^13^C/^12^C) and grams of carbon per gram rock (from the average organic carbon percentage of the sample set; averages and 1 standard deviation reported in **Table 1**), assuming no change in the quantity of organic matter between time points. The rate of change, expressed as nmol C incorporated g^-1^_rock_ d^-1^, was then calculated by dividing the mass change by the time period and correcting for the ratio of ^13^C-label in the substrate pool. Thus, the degree of incorporation, or isotopic composition, will depend on the moles of labeled carbon incorporated into biomass and the pre-existing quantity of organic carbon. Due to very small and inconsistent changes in the carbon ratio as well as very low organic carbon content in the subsurface North Pond basalts (as described below, and see **Table 1**), rates were only calculated for seafloor-exposed basalts. Furthermore, although the instrument precision was established as 0.3‰, rates of change were conservatively assumed to be robust if there was more than a 2‰ difference between samples; values less than 2‰ difference are reported as below the detection limit (b.d.l.).

### DNA Extraction and Quantitative Polymerase Chain Reaction (qPCR) Analysis of Genes

DNA was extracted from aliquots of the incubated basalts from the JdF2009, Ula Nui and Marker 2 samples that had been stored frozen (-80°C) until analysis. Upon thawing on ice, roughly 0.5–1 g of basalt fragments were transferred via a sterile spatula and roughly ground in a sterilized mortar. DNA was extracted from roughly 0.5 g of this material using the PowerSoil^®^ DNA Isolation Kit (MO BIO Laboratories, Inc.) with the following modifications to the manufacturer’s protocol: the cell lysis procedure consisted of two rounds of heating the sample (80°C for 5 min) and then bead beating (6 m/s for 60 s on a FastPrep-24^TM^ Instrument, MP Biomedicals). DNA was eluted in 100 μL of solution C6 (10 mM Tris) according to the manufacturer protocols. DNA concentrations were determined by fluorometry using the Qubit dsDNA^®^ HS Assay kit (Life Technologies Corporation) following the manufacturer instructions.

The abundance of taxonomic marker (i.e., 16S rRNA gene) and various functional genes for carbon cycling pathways was quantified by qPCR using the primer combinations outlined in **Table 2**. QPCR was performed on a Stratagene MX3005P using the iTaq Universal SYBR Green mix (Biorad Inc.). Bacterial 16S rRNA genes, RuBisCO form II enzyme genes of the reductive pentose phosphate cycle/Calvin-Benson-Bassham cycle (*cbbM*), methyl coenzyme M reductase genes of the reductive acetyl-CoA pathway (*mcrA*), and ATP citrate lyase genes of the reverse TCA cycle (*aclB*) were successfully amplified. Attempts to amplify archaeal 16S rRNA genes with multiple primer sets (**Table 2**) were not successful. Appropriate standard and negative controls were constructed from StrataClone vectors (Agilent Technologies), following manufacturer protocols, and linearized using a Hind III (New England Biolabs) restriction digest. QPCR protocols consisted of 180 s denaturation at 94°C, followed by 40 cycles of 60 s at 94°C, 60 s at the annealing temperature (**Table 2**), 45 s at 72°C, and 15 s at 80°C, at the end of which fluorescence was measured. The efficiencies of all reactions were between 96 and 103%. For the *mcrA* gene analyses, the amplicon size was verified.

**Table 2:** Oligonucleotide primers and conditions used in this study for quantitative PCR analysis.

Category	Target gene	Forward primer (nM)	Reverse primer (nM)	Positive control	Ta	Reference
Calvin Benton Bassham	*RuBisCO* form II	*cbbM591F* (200) TTC TGG CTG GGB GGH GAY TTY ATY AAR AAY GAC GA	*cbbM918R* (200) CCG TGR CCR GCV CGR TGG TAR TG	*Thiomicrospira crunogena* (DMS No. 12353)	55	Campbell and Cary (2004)
*rTCA*	*ATP citrate lyase*	*aclB892F (200)* TGG ACM ATG GTD GCY GGK GGT	*aclB1204R* (200) GTT GGG GCC RCC WCK KCK NAC	*Sulfurovum denitrificans* (DMS No. 1251)	55	Takai et al. (2005)
Methano genesis	*Methyl CoM reductase*	*qmcrAF-alt (150)* GAR GAC CAC TTY GGH GGT TC	*ML-R (200)* TTC ATT GCR TAG TTW GGR TAG TT	*Methanosarcina acetivorans, Methanococcus jannaschii*	55	Luton et al. (2002), Ver Eecke et al. (2012)
*Bacteria*	16S rRNA	*Bact1369F* (1000) GTT GGG GCC RCC WCK KCK NAC	*Prok1541R* (1000) CGG TGA ATA TGC CCC TGC	*Arcobacter nitrofigulis*	59	Suzuki et al. (2001)
*Archaea*	16S rRNA	*Arch1-1369F* (500) CGG TGA ATA CGT CCC TGC *+ Arch2-1369F* (500) *CGG TGA ATA TGC CCC TGC*	*Prok1541R* (1000) CGG TGA ATA TGC CCC TGC	*Ferroplasma acidarmonas Fer1*	59	Suzuki et al. (2001)
*Archaea*	16S rRNA	*Arch349F (500)* GYG CAS CAG KCG MGA AW	*Arch806R (500*) GGA CTA CVS GGG TAT CTA AT	*Ferroplasma acidarmonas Fer1*	54	Takai and Horikoshi (2000)

*Ta, annealing temperature during QPCR reaction*.

### Screening of Microbial Genes Associated with the Potential to Fix Carbon

Although 16S rRNA gene sequencing was beyond the scope of the present study, a parallel study determined the bacterial community composition of the same two Loihi basalts based on Ion Torrent sequencing of the V6 hypervariable region of the 16S rRNA gene (Jacobsen Meyers et al., 2014). To determine which of the bacterial orders recovered from the two Loihi basalts were potentially capable of carbon fixation via the pathways targeted with the qPCR analysis, we searched the Integrated Microbial Genomes (IMG) database (Markowitz et al., 2006) for genomes within the orders previously detected on these samples (Jacobsen Meyers et al., 2014) using the “Find Functions” search and the “Enzymes (list)” filters. EC 4.1.1.39 was used to search for the *cbbM* gene, and EC 2.3.3.8 was used to search for the *aclB* genes (analysis done 07-09 October 2014). Genomes were scored as positive if they contained either the *cbbM* or *aclB* gene, and then the number of genes within the order was tallied to calculate the percentage of genomes within that order capable of producing each enzyme. This analysis depends on the assumptions that: (a) if an organism is a member of a clade in which a specific enzyme is found more frequently in genomes from that clade, then that organism is more likely to have the enzyme, (b) the annotations in IMG are reliable.

## Results

### ^13^C-Enrichment of Organic Matter

The average organic carbon content of the basalts used in this study was quite low, in all cases less than 0.1 weight percent, and with several less than 0.02 weight percent (**Tables 1** and **3**). These values are lower than has been measured for North Pond basalts previously based on loss-on-ignition measurements (Expedition 336 Scientists, 2012c,d). It is probable that the acid-washing step to remove unincorporated ^13^C-bicarbonate resulted in the physical removal of some organic matter. If so, we posit that it did not impact the carbon isotope ratio of the residual material, as bulk erosion of a biofilm is not likely to selectively remove organic constituents. Highly oxidized basalt (i.e., Ula Nui, Marker 2, J2-626-R1, U1382A 3R4, U1382A 7R2) had slightly higher organic carbon content (average ± 1 standard deviation of 0.050 ± 0.028 wt %, *n* = 42 samples) than the glassy basalt samples (0.027 ± 0.010, *n* = 14 samples), which is consistent with previous observations of higher organic carbon in altered basalt from North Pond (Expedition 336 Scientists, 2012c,d). These low values required sensitive detection methods for determining stable isotopic composition of organic carbon by IRMS. A few samples (*n* = 5 out of 56 total) did not have enough carbon to enable a robust isotopic determination; these samples are not included in the following analyses.

**Table 3:** Summary of organic carbon content and stable carbon isotope composition of seafloor basalt samples after incubation.

ID	Time (h)	OC wt %	δ^13^C-OC (‰)	(^13^C/^12^C) × 10^2^	Δ ^13^C/^12^C	Δ ng ^13^C/g_rock_	Δ nmol C/g_rock_/d
JdF2009	*T_0_*	*0.040*	*-19.5*	*1.096*	*n.a.*	*n.a.*	*n.a.*
	1.25	0.032	-21.4	1.094	b.d.l.	–	–
	1.25	0.034	-21.1	1.094	b.d.l.	–	–
	23.75	0.028	-19.5	1.096	b.d.l.	–	–
	23.75	0.022	-7.2	1.110	1.4 × 10^-4^	41	12.7
	23.75	0.027	-19.9	1.096	b.d.l.	–	–
	139.75	0.023	6.1	1.125	2.9 × 10^-4^	86	4.5
	139.75	0.043	-17.9	1.098	1.9 × 10^-5^	6	0.3

Ula Nui	*T_0_*	*0.044*	*-20.4*	*1.095*	*n.a.*	*n.a.*	*n.a.*
	1	0.113	-22.1	1.093	b.d.l.	–	–
	1	0.061	-21.2	1.094	b.d.l.	–	–
	1	0.041	-16.3	1.100	4.6 × 10^-5^	27	92.5
	24.25	0.050	-17.2	1.099	3.5 × 10^-5^	21	3.0
	24.25	0.059	-19.6	1.096	b.d.l.	–	–
	24.25	0.040	-20.1	1.095	b.d.l.	–	–
	124.25	0.043	-19.9	1.096	b.d.l.	–	–
	124.25	0.057	-13.6	1.103	7.6 × 10^-5^	44	1.2
	124.25	0.074	-18.2	1.098	2.5 × 10^-5^	15	0.4

Marker 2	*T_0_*	*0.043*	*-21.1*	*1.094*	*n.a.*	*n.a.*	*n.a.*
	1	0.078	-21.4	1.094	b.d.l.	–	–
	1	0.086	-16.1	1.100	5.7 × 10^-5^	33	114.3
	24	0.035	-22.1	1.093	b.d.l.	–	–
	24	0.042	-19.1	1.097	2.3 × 10^-5^	13	1.9
	24	0.063	-19.7	1.096	b.d.l.	–	–
	124.25	0.029	-9.3	1.108	1.3 × 10^-4^	77	2.2
	124.25	0.052	-10.5	1.106	1.2 × 10^-4^	69	1.9
	124.25	0.095	-10.8	1.106	1.2 × 10^-4^	67	1.9

J2-626-R1	*T_0_*	*0.027*	*-22.9*	*1.092*	*n.a.*	*n.a.*	*n.a.*
	118.5	0.032	-16.3	1.100	7.4 × 10^-5^	39	1.0
	118.5	0.012	-6.1	1.111	1.9 × 10^-4^	100	2.4
	118.5	0.027	-19.1	1.097	4.2 × 10^-5^	23	0.6
	237	0.102	14.3	1.134	4.2 × 10^-4^	220	2.7
	237	0.032	-16.0	1.100	7.7 × 10^-5^	41	0.5
	237	0.105	49.1	1.173	8.0 × 10^-4^	426	5.2
	480	n.d.	82.6	1.210	1.2 × 10^-3^	625	3.8
	480	0.101	51.1	1.175	8.3 × 10^-4^	438	2.6
	480	0.043	54.8	1.179	8.7 × 10^-4^	461	2.8

J2-627-R3	118.5	0.024	-18.0	1.098	b.d.l.	–	–
	118.5	0.027	22.6	1.143	4.5 × 10^-4^	109	2.7
	118.5	0.020	58.1	1.183	8.5 × 10^-4^	204	5.0
	237	0.020	-0.1	1.118	2.0 × 10^-4^	48	0.6
	237	0.022	27.2	1.148	5.1 × 10^-4^	121	1.5
	237	0.019	9.3	1.128	3.1 × 10^-4^	73	0.9
	480	0.019	32.4	1.154	5.6 × 10^-4^	135	0.8
	480	0.021	15.6	1.135	3.8 × 10^-4^	90	0.5
	480	0.038	30.5	1.152	5.4 × 10^-4^	130	0.8

*b.d.l., below detection limit; n.a., not applicable; n.d., not determined. See text for details.*

**FIGURE 2:**
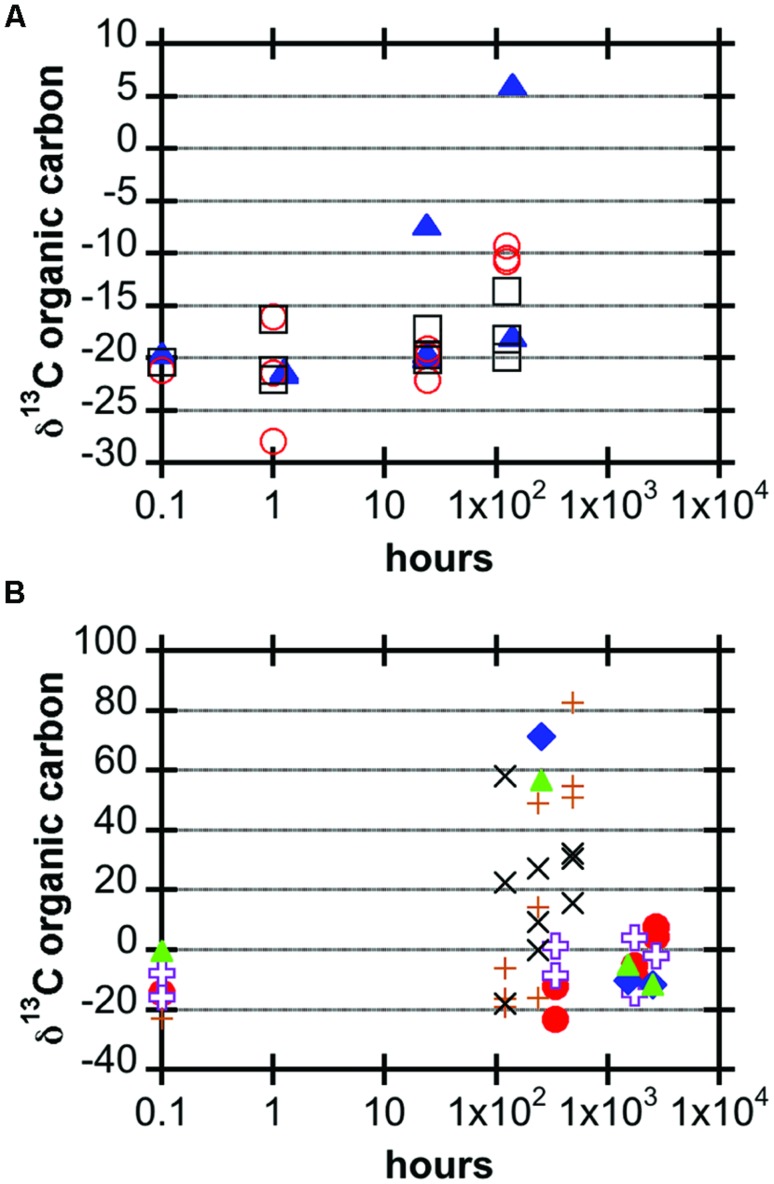
**Carbon stable isotopic composition of organic matter in basalt samples after incubation, expressed in the delta notation of per mil (note logarithmic time scale). (A)** JdF2009 (blue filled triangles), Ula Nui (black open squares), and Marker 2 (red open circles). **(B)** U1382A 3R4 (purple open crosses), U1382A 7R2 (red filled circles), U1383C 13R1 (blue filled diamonds), U1383C glass (green filled triangles), J2-626-R1 (brown plus signs), J2-627-R3 (black x’s).

The JdF2009, Marker 2, and Ula Nui samples, which were incubated for up to a week, all showed some degree of ^13^C enrichment of organic matter over time (**Figure 2A**; **Table 3**). The JdF2009 glassy basalt had the largest heterogeneity in isotopic values between samples, which may have been affected by the relatively low organic carbon concentration in this sample set (**Tables 1** and **3**), allowing minor amounts of carbon uptake to skew the isotopic ratio to a greater degree than in samples with higher organic carbon pools. Some of the North Pond basalts, which were incubated for a much longer period of time, showed significant incorporation of ^13^C into organic matter over time, while others did not exhibit a strong trend (**Figure 2B**). The two seafloor-exposed North Pond rocks—J2-626-R1 and J2-627-R3—had the highest incorporation of ^13^C into organic matter by the end of the incubations. The glassy subsurface basalts from Hole U1383C—U1383C glass and U1383C 13R1—also exhibited some incorporation of ^13^C into organic matter over time, whereas the massive basalts from Hole U1382A did not show significant incorporation of ^13^C over time. Again, the degree of incorporation in the glassy basalts could have been skewed by the relatively low organic carbon concentration of these samples. However, it should be noted that some of the Hole U1383C glassy basalts had positive signs of contamination based on fluorescent microsphere tracer tests, and it is possible the contaminant organisms could have been responsible for some degree of carbon uptake.

**FIGURE 3:**
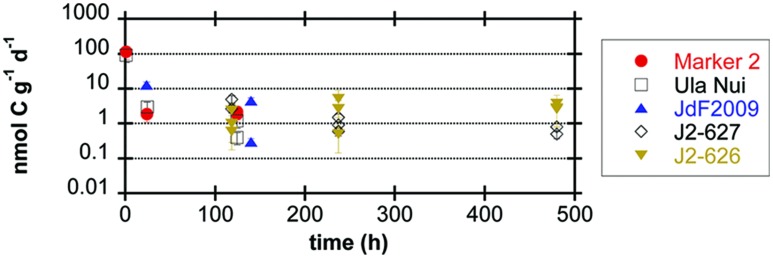
**Potential rates of carbon fixation by seafloor-exposed basalt-hosted microbial communities (expressed as nmol C incorporated g^-1^_rock_ d^-1^) as a function of incubation time, based on incubation experiments of basalt samples with ^13^C-labeled bicarbonate.** Error bars represent the range of the coefficient of variance from the measurement of organic carbon weight percent, since the mean value for each sample set was applied to all samples in that set (see Materials and Methods); in some cases, data symbol is larger than the error bar range. Samples with isotopic composition differences of less than 2‰ from the time zero sample (**Table 3**) were assumed to be below the limit of detection of the method (see Materials and Methods) and are not reported here.

Conversion of carbon isotope ratios into potential rates of carbon fixation revealed that all of the seafloor-exposed rocks supported potential carbon fixation rates in the range of 0.1–10 nmol C g^-1^_rock_ d^-1^, with the samples from the Loihi seamount having a few outliers with rates near 100 nmol C g^-1^_rock_ d^-1^ from samples incubated for a relatively short period of time (**Figure 3**; **Table 3**). However, four of the five seafloor-exposed basalt incubations contained at least one sample that did not have detectable incorporation of ^13^C-label into organic matter during the course of the incubations (**Table 3**), based on a conservative requirement of a 2‰ or greater difference between the time zero and samples to define a change in isotopic composition. This emphasizes the heterogeneity of the organic carbon content and likely of the biofilm distributions between samples in the incubations. It should be noted that the relatively low organic carbon content in the samples affects the calculation of potential rates and represents a potentially low end-member if some of the organic carbon was lost during acid-washing steps.

### Microbial Abundance and Functional Potential

DNA was successfully extracted from roughly 0.5 g samples of seafloor-exposed basalts from the JdF2009, Marker 2, and Ula Nui samples, resulting in yields between 2 and 31 ng DNA g^-1^_rock_ (based on Qubit^®^dsDNA HS Assay concentration assays; data not shown). Quantitative PCR assays with high efficiencies were possible from the DNA extractions without any further purification steps.

**FIGURE 4:**
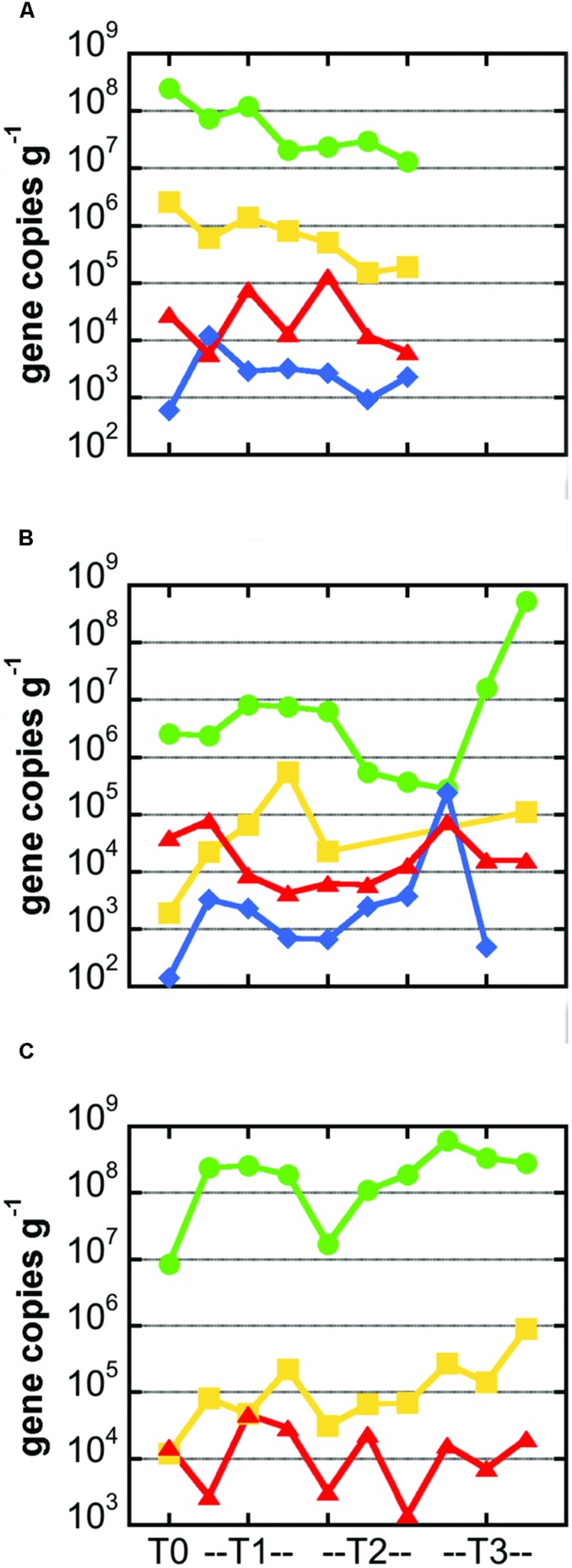
**Abundance of carbon cycling functional genes in microbial communities hosted in basalts samples from the Marker 2 (A), Ula Nui (B), and JdF2009 (C) sample sets.** In all panels, gene abundance for each sample is presented as number of gene copies per gram of rock. Green circles: bacterial 16S rRNA gene; yellow squares: RuBisCO form II gene *cbbM* from Calvin Benton Bassham pathway; red triangles, methyl coenzyme M reductase gene *mcrA* from methane cycling pathways; blue diamonds, ATP citrate lyase gene *aclB* from reverse TCA process. Note missing symbols in **(A)** indicate samples that were not available for analysis, while missing symbols in **(B,C)** indicate values that were not above the limit of detection (∼10^3^ gene copies per gram for *cbbM* and ∼10^2^ gene copies per gram for *aclB*). Lines connecting symbols are for esthetic purpose and convey no meaning.

Bacterial 16S rRNA gene copy numbers per gram of basalt ranged from roughly 10^7^–10^9^ for the Marker 2 and JdF2009 incubations, with lower values of 10^5^–10^7^ for all but two of the Ula Nui incubations (**Figure 4**). These gene copy densities are quite consistent with those reported for other seafloor exposed basalts (Santelli et al., 2008). There was no apparent increase or decrease in cell density between the sample time points, as time point replicates displayed similar ranges of variations as between time points. Assuming roughly four copies of the 16S rRNA gene per cell, these values would translate to approximately 10^6^–10^8^ cells g^-1^_rock_ for the Marker 2 and JdF2009 basalts, and 10^5^–10^6^ cells g^-1^_rock_ for the Ula Nui basalt. These values are in the same range as those reported for the Ula Nui and Marker 2 basalts in another study (Jacobsen Meyers et al., 2014), although the earlier study did not demonstrate a higher cell density on the Marker 2 samples versus the Ula Nui samples as is potentially evident here. In the present study, archaeal 16S rRNA genes could not be quantified. A previous study documented that archaea made up only 1–2% of the total microbial community on Ula Nui and Marker 2 basalts (Jacobsen Meyers et al., 2014), which is consistent with other studies of seafloor-exposed basalt microbial communities (Santelli et al., 2008).

Genes involved in carbon fixation pathways were detected in all of the samples analyzed, although in different relative amounts (**Figure 4**). The RuBisCO form II enzyme gene *cbbM* was predominantly the most abundant carbon fixation gene in all of the samples, often by several orders of magnitude (**Figure 4**). The *cbbM* gene was most abundant in the Marker 2 basalt samples, ranging from 10^5^ to 10^7^ *cbbM* gene copies g^-1^_rock_ (**Figure 4**). Notably, this gene was less abundant in the JdF2009 basalt samples (10^4^–10^6^ gene copies g^-1^_rock_). The abundance of the *cbbM* gene in the Ula Nui samples was erratic, ranging from below detection limit (<1000 gene copies g^-1^_rock_) to nearly 10^6^ gene copies g^-1^_rock_. Although archaeal 16S rRNA genes were not detected, methyl coenzyme M reductase (*mcrA* gene) was detected in all of the samples at 10^3^–10^5^ gene copies g^-1^_rock_ (**Figure 4**), and the size of the amplicon matched the expected product size of the primer set (∼265 bp). The *aclB* gene for ATP citrate lyase was observed in the Marker 2 and Ula Nui basalt samples but was undetectable in the JdF2009 basalt samples. This gene was consistently abundant at 10^3^–10^4^ gene copies g^-1^_rock_ in the Marker 2 samples, but ranged from 10^2^ to 10^6^ gene copies g^-1^_rock_ in the Ula Nui samples.

**FIGURE 5:**
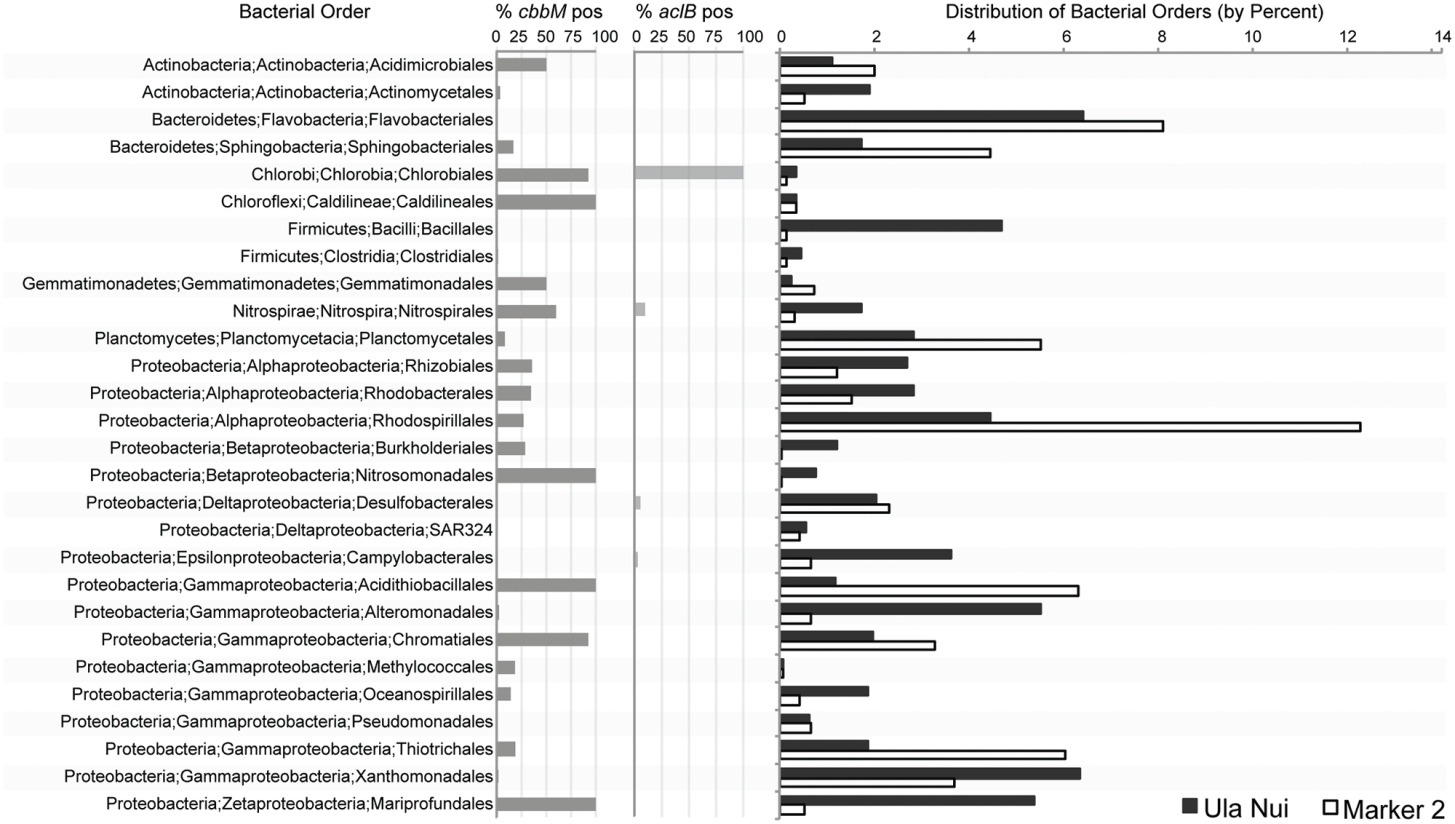
**Percent abundance of bacterial orders detected by Ion Torrent sequencing of the V6 region of the 16S rRNA gene from environmental DNA extracts from the Ula Nui and Marker 2 Loihi seafloor basalts versus the percent abundance of publically available genomes from the same orders with evidence of *cbbM* or *aclB* genes.** Percent abundance of bacterial orders has been published previously (Jacobsen Meyers et al., 2014) and is used here with permission.

We determined the bacterial orders putatively responsible for the carbon fixation detected on the Ula Nui and Marker 2 basalts by searching the IMG database (Markowitz et al., 2006) for the presence of *cbbM* and *aclB* in sequenced genomes from all bacterial orders previously detected on these samples (**Figure 5**), using a similar approach as reported elsewhere (Jacobsen Meyers et al., 2014). The presence of *cbbM* was distributed widely in the bacterial orders detected, while *aclB* was detected only in the orders Chlorobiales, Nitrospirales, Desulfobacterales, and Campylobacterales. Although *cbbM* is present in many orders, it is common (present in ≥50% of the genomes queried) in only a few orders: Acidimicrobiales, Chlorobiales, Caldilineales, Gemmatimonadales, Nitrospirales, Nitrosomonadales, Acidithiobacillales, Chromatiales, and Mariprofundales. Of these, Acidimicrobiales, Nitrospirales, Acidithiobacillales, Chromatiales, and Mariprofundales are the most abundant orders, indicating that these groups potentially contribute most significantly to overall rates of basalt carbon fixation in the Loihi basalts, assuming equal rates for all active groups. The presence of *aclB* was common in the Nitrospirales and Chlorobiales.

## Discussion

Using stable carbon isotope incubation experiments, we demonstrate autotrophic carbon fixation by basalt-hosted biofilm communities, with consistent potential carbon fixation rates of 0.1–10 nmol C g^-1^_rock_ d^-1^ in the seafloor-exposed basalts examined in this study (**Figures 2** and **3**; **Table 3**). Notably, subsurface basalts exhibit markedly lower and/or undetectable carbon assimilation (**Figure 2**). The potential rates of carbon fixation are similar to or lower than recently available measurements of total community microbial activity on seafloor-exposed basalts (Jacobsen Meyers et al., 2014). This suggests that the heterotrophic incorporation of exogenous organic carbon may be playing a significant role in this habitat, which is consistent with recent observations of depleted organic carbon content in subsurface crustal fluids as compared to bottom seawater (Lin et al., 2012). Nevertheless, when scaling the potential carbon fixation rates measured in this study to the global reservoir of the oceanic crustal environment, these results support earlier predictions on the magnitude of carbon fixation in oceanic crust (Bach and Edwards, 2003), as discussed below. We note that the rates presented here do not preclude the possibility of elevated rates of basalt-hosted autotrophy *in situ*, nor do they definitively establish the relative proportions of autotrophy versus heterotrophy among deep-sea basalts. Finally, the Calvin cycle appears to be the dominant autotrophic process used in seafloor-exposed basalt based on gene abundance (**Figure 4**). Based on the presence of these genes in microbial groups common on seafloor-exposed basalts (**Figure 5**), we suggest that bacteria in the orders Acidimicrobiales, Acidithiobacillales, Chromatiales, and Mariprofundales are potentially the most active carbon-fixing bacteria in seafloor-exposed basalts.

### Carbon Fixation Potential and Rates

Our stable carbon isotope experiments provide the first empirical documentation of potential carbon fixation by basalt-hosted microbial communities (**Figures 2** and **3**; **Table 3**). Potential carbon fixation rates of 0.1–10 nmol C g^-1^_rock_ d^-1^ were consistently observed under oxic conditions for seafloor basalts sourced from several deep-sea environments (**Figure 3**; **Table 3**). Higher potential rates near 100 nmol C g^-1^_rock_ d^-1^ were calculated for some of the shorter (1 h) seafloor-exposed basalt incubations, while the 0.1–10 nmol C g^-1^_rock_ d^-1^ range was fairly consistent in longer incubation periods. This may suggest diminishing carbon fixation potential influenced by incubation effects such as decreased pressure from *in situ* conditions, decreased oxidant availability, nutrient limitation, or other factors. High potential rates may also reflect the impact of relatively small additions in ^13^C-labeled molecules into very organic poor samples having a strong impact on the overall isotopic composition of the sample. Conversely, four out of the five seafloor-exposed basalts also contained at least one sample that exhibited no detectable shift in carbon isotopic composition over the course of incubation (based on a conservative limit of 2‰ or greater isotopic change; **Table 3**). This likely reflects patchiness and heterogeneity of biofilm distribution in the pieces of basalt used in the incubation.

Notably, these potential carbon fixation rates are similar to or smaller than the maximum potential rates of extracellular enzyme activity measured for basalts from the Loihi seamount, which ranged from 10 to 1,500 nmol_substrate_ g^-1^_rock_ d^-1^ (Jacobsen Meyers et al., 2014). However, higher enzyme activities are to be expected for several reasons. First, the two enzymes assayed—alkaline phosphatase and leucine aminopeptidase—are present in both autotrophic and heterotrophic lineages, and therefore the community exoenzyme pool is likely larger than the community pool of proteins involved in autotrophic carbon fixation. Second, the exoenzyme assays were conducted at saturating substrate concentrations, leading to maximum potential rates, while the ^13^C-bicarbonate assays analyzed here were not at saturation. Third, carbon fixation by basalt biofilm microbial communities is related to anabolic metabolic reaction whereas the extracellular enzyme assays are related to catabolic processes. Finally, the possibility for loss of biofilms from the acid-washing steps used to prepare the samples may have lead to underestimates of the potential carbon fixation rates.

Scaling the potential rates of carbon fixation measured here (range of 0.1–10 nmol C g^-1^_rock_ d^-1^ with outliers of ∼100 nmol C g^-1^_rock_ d^-1^; **Figure 3**; **Table 3**) to the size of the surface crustal reservoir as calculated elsewhere (Bach and Edwards, 2003), we extrapolate that roughly 10^9^–10^12^ g C year^-1^ could be fixed in this ecosystem, which matches the earlier predictions of 10^11^–10^12^g C year^-1^ based on thermodynamic and bioenergetics considerations (Bach and Edwards, 2003). The match between empirical measures of potential microbial activity in oceanic crust and theoretical estimations provides support that the potential rates measured here are indeed reflective of processes in the environment. Moreover, the overlap between measured and theoretical values further supports the hypothesis that water-rock reactions can support significant microbial life in ridge flank hydrothermal systems (Bach and Edwards, 2003).

Potential carbon fixation rates were generally below detection in subseafloor basalts (**Figure 2**), suggesting (1) that autotrophy is not a dominant metabolic pathway in the subsurface; (2) that bulk rates are too low to detect, regardless of their dominance in the ecosystem; or (3) that incubation conditions were not conducive to activity as compared to *in situ* conditions (such as lower pressure; Nagata et al., 2010). Another possible complicating factor is that these incubations were performed in oxic seawater. While the North Pond crustal subsurface is oxic (Orcutt et al., 2013), warranting the experimental approach, it is possible that inclusion of anoxic incubations may have increased the total rate. The presence of *mcrA* genes in our analysis of seafloor rocks (**Figure 4**) indicates that some anoxic carbon fixation is possible in anaerobic microniche within the rocks. Recent work demonstrates that anoxic, hydrothermal subsurface crustal fluids are depleted in organic carbon compared to bottom seawater, suggesting that the subsurface is net heterotrophic and reliant on organic carbon from outside (Lin et al., 2012). By contrast, however, fluids from the same anoxic system revealed indications of autotrophic production of dissolved organic carbon (McCarthy et al., 2011). The balance of autotrophy versus heterotrophy in the crustal biosphere is yet to be resolved.

### Microbial Groups Involved in Carbon Fixation on Basalts

The RuBisCO form II *cbbM* gene was the most abundant carbon fixation gene in the all of the samples investigated (**Figure 4**), as compared to methyl coenzyme M reductase *mcrA* gene or the *aclB* gene for ATP citrate lyase, suggesting that the Calvin cycle is the dominant pathway for carbon fixation in these basalt-hosted biofilms. Bacteria within the phylum Nitrospirae, genus *Nitrospira*, and the Gammaproteobacterial order Chromatiales, are recovered consistently from seafloor-exposed basalts (Mason et al., 2009; Santelli et al., 2009; Sylvan et al., 2013) and were present on the Loihi basalts used in this study (Jacobsen Meyers et al., 2014). The majority of sequenced genomes from these groups contain *cbbM* genes (**Figure 5**). This indicates they may participate in autotrophic carbon fixation and are potentially important primary producers on seafloor-exposed basalts. In the case of Nitrospirales, however, the *cbbM* gene is a form IV version of the protein not functional in carbon fixation (Goltsman et al., 2009; Lücker et al., 2010), and this gene may instead be used for sulfur metabolism (Ashida et al., 2005). Likewise, the orders Acidimicrobiales, Acidithiobacillales, and Mariprofundales were also abundant on the Loihi samples, are likely to contain *cbbM* genes, and are therefore likely significant contributors to basaltic carbon fixation as well. Acidimicrobiales is an order within the Actinobacteria into which the Ocean Crust Clade XIII falls; this group consists of organisms detected only in basalt samples (Mason et al., 2009). This suggests that these putative carbon fixers are likely widespread on seafloor-exposed basalts. Acidithiobacillales and Mariprofundales have not previously been detected on seafloor basalts (Jacobsen Meyers et al., 2014), possibly due to the shallow sequencing depth typical of clone libraries (Santelli et al., 2008, 2009; Mason et al., 2009; Sylvan et al., 2013).

The only bacterial order detected on Loihi basalts that has a high percentage of genomes containing *aclB* genes is the Chlorobiales (**Figure 5**), although this group is generally not present in high abundances on basalts (as cited above) and thus likely plays a minor role. While only a small proportion of Campylobacterales genomes encode for *aclB* (**Figure 5**), the Campylobacterales genera most likely to be detected on basalts (i.e., *Sulfuricurvum*, *Sulfuriminas*, *Thiovulum*, and *Nitratifractor*) all contain the rTCA cycle. However, the vast majority of sequenced genomes from this order belong to *Campylobacter* and *Helicobacter*, which are of medical interest but do not carry out carbon fixation via the rTCA cycle. Similarly, while only a small percentage of Nitrospirales genomes contain *aclB* (**Figure 5**), we posit that basalt-hosted Nitrospirales are also likely fixing carbon via the rTCA pathway based on two arguments. First, while members of the genus *Nitrospira* are the only members of the Nitrospirales that contain *aclB* (Lücker et al., 2010), members of the genus *Leptospirillum* seem to universally have an alternative pathway to cleave citrate using citryl-CoA synthetase and citryl-CoA lyase (Levicán et al., 2008; Goltsman et al., 2009). Therefore, two the three genera within the order Nitrospirales fix carbon using the rTCA cycle (the third genus, *Thermodesulfovibrio*, are heterotrophs). Second, out of the three genera in the order, *Nitrospira* are common in marine systems (Lücker et al., 2010) while the other two are not; therefore, the Nitrospirales on basalts are likely to be *Nitrospira*. It should also be noted that the order Desulfobacterales, which was abundant on the Loihi basalts (**Figure 5**), likely utilizes the acetyl-CoA pathway for carbon fixation (Kuever et al., 2005), but that was not tested here.

Interestingly, *mcrA*, which is associated with the anaerobic production and consumption of methane, was detected in all three incubations assayed (**Figure 4**), even though the incubations were conducted under oxic conditions. Despite the difficulty in detecting Archaea based on the 16S rRNA gene, the presence of methyl coenzyme M reductase indicates that Archaea were present in seafloor-exposed basalts. Two lines of evidence indicate that, while methane cycling may not be a quantitatively large metabolic process on seafloor-exposed basalts (although this remains to be tested), methanogenesis is likely happening to some degree on seafloor and subseafloor basalts. First, *mcrA* genes were detected on seafloor (Mason et al., 2009) and subseafloor (Lever et al., 2013) basalts from the Juan de Fuca Ridge and ridge flank. Second, incubations with seafloor and subseafloor basalts resulted in production of measurable methane (Lysnes et al., 2004; Lever et al., 2013). The presence of *mcrA* genes, indicative of an anaerobic process, indicates that there are anaerobic microniches in the pits and pockets of the basalt rocks, even when exposed to oxic seawater, as posited previously (Edwards et al., 2005) and owing to the presence of genes related to methanogenesis, denitrification, and sulfate reduction on basalt from the Juan de Fuca Ridge (Mason et al., 2009).

This stable carbon isotope study provides empirical evidence for the potential for carbon fixation by basalt-hosted microbial communities in the deep-sea. Though local carbon fixation rates are small in comparison to highly productive photosynthetic regions, the globally scaled rates of 10^9^–10^12^ g C fixed per year nevertheless indicate autotrophy as an important metabolism in the oceanic crust and the broader deep-sea ecosystem. These rates of net production are especially important in the dark ocean, which is devoid of photosynthesis. This study does not definitely identify which basalt biofilm microorganisms are involved in carbon fixation, yet functional gene abundance suggests that the Calvin cycle is the primary biochemical mechanism used by basalt microbial communities to fix carbon. While potential carbon fixation rates were quantifiable for seafloor-exposed rocks, carbon fixation activity in deeper basalts was not resolvable with the approach used in this study and warrants further investigation, as the size of the subsurface crustal reservoir is significantly larger than seafloor-exposed basalts.

## Conflict of Interest Statement

The authors declare that the research was conducted in the absence of any commercial or financial relationships that could be construed as a potential conflict of interest.

## References

[B1] Alt J. C. Mata P. (2000). On the role of microbes in the alterations of submarine basaltic glass: a TEM study. *Earth Planetary Sci. Lett.* 181 301–313. 10.1016/S0012-821X(00)00204-1

[B2] Alt J. C. Teagle D. A. H. (1999). The uptake of carbon during alteration ocean crust. *Geochim. Cosmochim. Acta* 63 1527–1535. 10.1016/S0016-7037(99)00123-4

[B3] Ashida H. Danchin A. Yokota A. (2005). Was photosynthetic RuBisCO recruited by acquisitive evolution from RuBisCO-like proteins involved in sulfur metabolism? *Res. Microbiol.* 156 611–618. 10.1016/j.resmic.2005.01.014 15950120

[B4] Bach W. Edwards K. J. (2003). Iron and sulfide oxidation within the basaltic ocean crust: implications for chemolithoautotrophic microbial biomass production. *Geochim. Cosmochim. Acta* 67 3871–3887. 10.1016/s0016-7037(00)00304-301

[B5] Bach W. Humphris S. Fisher A. T. (2004). Fluid flow and fluid-rock interaction within ocean crust: reconciling geochemical, geological, and geophysical observations. *Am. Geophys. Union* 144 99–117.

[B6] Campbell B. J. Cary S. C. (2004). Abundance of reverse tricarboxylic acid cycle genes in free-living microorganisms at deep-sea hydrothermal vents. *Appl. Environ. Microbiol.* 70 6282–6289. 10.1128/AEM.70.10.6282-6289.2004 PMC522104 15466576

[B7] Edmond J. M. Von Damm K. L. (1983). Hot springs on the ocean floor. *Sci. Am.* 248 78–93. 10.1038/scientificamerican0483-78

[B8] Edwards K. J. Bach W. Mccollom T. M. (2005). Geomicrobiology in oceanography: microbe–mineral interactions at and below the seafloor. *Trends Microbiol.* 13 449–456. 10.1016/j.tim.2005.07.005 16054363

[B9] Edwards K. J. Mccollom T. M. Konishi H. Buseck P. R. (2003a). Seafloor bioalteration of sulfide minerals: results from in-situ incubation studies. *Geochim. Cosmochim. Acta* 67 2843–2856. 10.1016/S0016-7037(03)00089-9

[B10] Edwards K. J. Rogers D. R. Wirsen C. O. Mccollom T. M. (2003b). Isolation and characterization of novel, psychrophilic, neutrophilic, Fe-oxidizing, chemolithoautotrophic alpha- and gamma-Proteobacteria from the deep sea. *Appl. Environ. Microbiol.* 69 2906–2913. 10.1128/AEM.69.5.2906-2913.2003 PMC154528 12732565

[B11] Expedition 336 Scientists. (2012a). “Methods,” in *Proceedings of the Integrated Ocean Drilling Program* Vol. 336 eds Edwards K. J. Bach W. Klaus A. Expedition 336 Scientists (Tokyo: Integrated Ocean Drilling Program Management International, Inc.). 10.2204/iodp.proc.336.102.2012

[B12] Expedition 336 Scientists. (2012b). “Sediment and basement contact coring,” in: *Proceeding of the Integrated Ocean Drilling program* Vol. 336 eds Edwards K. J. Bach W. Klaus A. Expedition 336 Scientists (Tokyo: Integrated Ocean Drilling Program Management International, Inc.).

[B13] Expedition 336 Scientists. (2012c). “Site U 1382,” in *Proceedings of the Integrated Ocean Drilling Program* Vol. 336 eds Edwards K. J. Bach W. Klaus A. Expedition 336 Scientists (Tokyo: Integrated Ocean Drilling Program Management International, Inc.).

[B14] Expedition 336 Scientists. (2012d). “Site U 1383,” in *Proceedings of the Integrated Ocean Drilling Program* Vol. 336 eds Edwards K. J. Bach W. Klaus A. Expedition 336 Scientists (Tokyo: Integrated Ocean Drilling Program Management International, Inc.).

[B15] Fisher A. T. Becker K. (2000). Channelized fluid flow in oceanic crust reconciles heat-flow and permeability data. *Nature* 403 71–74. 10.1038/47463 10638753

[B16] Goltsman D. S. Denef V. J. Singer S. W. Ver Berkmoes N. C. Lefsrud M. Mueller R. S. (2009). Community genomic and proteomic analyses of chemoautotrophic iron-oxidizing Leptospirillum rubrum (Group II) and Leptospirillum ferrodiazotrophum (Group III) bacteria in acid mine drainage biofilms. *Appl. Environ. Microbiol.* 75 4599–4615. 10.1128/AEM.02943-08 PMC2704813 19429552

[B17] Jacobsen Meyers M. E. Sylvan J. B. Edwards K. J. (2014). Extracellular enzyme activity and microbial diversity measured on seafloor exposed basalts from Loihi Seamount indicate importance of basalts to global biogeochemical cycling. *Appl. Environ. Microbiol.* 10.1128/AEM.01038-1014 PMC4135773 24907315

[B18] Kuever J. Rainey F. A. Widdel F. (2005). “Class IV. Deltaproteobacteria *class nov.*,” in *Bergey’s Manual of Systematic Bacteriology*, 2nd Edn, eds Brenner D. J. Krieg N. R. Staley J. T. (New York, NY: Springer-Verlag), 922–1144.

[B19] Lever M. A. Rouxel O. J. Alt J. C. Shimizu N. Ono S. Coggon R. M. (2013). Evidence for microbial carbon and sulfur cycling in deeply buried ridge flank basalt. *Science* 339 1305–1308. 10.1126/science.1229240 23493710

[B20] Levicán G. Ugaide J. A. Ehrenfeld N. Maass A. Parada P. (2008). Comparative genomic analysis of carbon and nitrogen assimilation mechanisms in three indigenous bioleaching bacteria: predictions and validation. *BMC Genomics* 9:581 10.1186/1471-2164-9-581 PMC2607301 19055775

[B21] Lin H.-T. (2013). *Biogeochemistry of Basement Fluids From the Sediment-Buried Juan de Fuca Ridge flanks.* Ph.D., University of Hawaii, Hilo, HI. 10.1038/ismej.2012.73 PMC3526168 22791235

[B22] Lin H.-T. Cowen J. P. Olson E. J. Amend J. P. Lilley M. D. (2012). Inorganic chemistry, gas compositions and dissolved organic carbon in fluids from sedimented young basaltic crust on the Juan de Fuca Ridge flanks. *Geochim. Cosmochim. Acta* 85 213–227. 10.1016/j.gca.2012.02.017

[B23] Lücker S. Wagner M. Maixner F. Pelletier E. Koch H. Vacherie B. (2010). A Nitrospira metagenome illuminates the physiology and evolution of globally important nitrite-oxidizing bacteria. *Proc. Natl. Acad. Sci. U.S.A.* 107 13479–13484. 10.1073/pnas.1003860107 PMC2922143 20624973

[B24] Luton P. E. Wayne J. M. Sharp R. J. Riley P. W. (2002). The mcrA gene as an alternative to 16S rRNA in the phylogenetic analysis of methanogen populations in landfill. *Microbiology* 148 3521–3530. 10.1099/00221287-148-11-3521 12427943

[B25] Lysnes K. Thorseth I. H. Steinsbu B. O. Øvreås L. Torsvik T. Pedersen R. B. (2004). Microbial community diversity in seafloor basalt from the Arctic spreading ridges. *FEMS Microbiol. Ecol.* 50 13–230. 10.1016/j.femsec.2004.06.014 19712362

[B26] Markowitz V. M. Korzeniewski F. Palaniappan K. Szeto E. Werner G. Padki A. (2006). The integrated microbial genomes (IMG) system. *Nucleic Acids Res.* 34 D344–D348. 10.1093/nar/gkj024 PMC1347387 16381883

[B27] Mason O. U. Di Meo-Savoie C. A. Van Nostrand J. D. Zhou J. Fisk M. R. Giovannoni S. J. (2009). Prokaryotic diversity, distribution, and insights into their role in biogeochemical cycling in marine basalts. *ISME J.* 3 231–242. 10.1038/ismej.2008.92 18843298

[B28] Mason O. U. Nakagawa T. Rosner M. Van Nostrand J. D. Zhou J. Maruyama A. (2010). First investigation of the microbiology of the deepest layer of ocean crust. *PLoS ONE* 5:e15399 10.1371/journal.pone.0015399 PMC2974637 21079766

[B29] McCarthy M. D. Beaupré S. R. Walker B. D. Voparil I. Guilderson T. P. Druffel E. R. M. (2011). Chemosynthetic origin of 14C-depleted dissolved organic matter in a ridge-flank hydrothermal system. *Nat. Geosci.* 4 32–36. 10.1038/ngeo1015

[B30] Nagata T. Tamburini C. Arístegui J. Baltar F. Bochdansky A. B. Fonda-Umani S. (2010). Emerging concepts on microbial processes in the bathypelagic ocean - ecology, biogeochemistry, and genomics. *Deep Sea Res. Part II Top. Stud. Oceanogr.* 57 1519–1536. 10.1016/j.dsr2.2010.02.019

[B31] Orcutt B. N. Bach W. Becker K. Fisher A. T. Hentscher M. Toner B. M. (2011a). Colonization of subsurface microbial observatories deployed in young ocean crust. *ISME J.* 5 692–703. 10.1038/ismej.2010.157 PMC3217339 21107442

[B32] Orcutt B. N. Sylvan J. B. Knab N. J. Edwards K. J. (2011b). Microbial ecology of the dark ocean above, at, and below the seafloor. *Microbiol. Mol. Biol. Rev.* 75 361–422. 10.1128/MMBR.00039-10 PMC3122624 21646433

[B33] Orcutt B. N. Wheat C. G. Rouxel O. J. Hulme S. Edwards K. J. Bach W. (2013). Oxygen consumption rates in subseafloor basaltic crust derived from a reaction transport model. *Nat. Commun.* 4 2539 10.1038/ncomms3539 24071791

[B34] Robador A. Jungbluth S. P. Larowe D. E. Bowers R. M. Rappé M. S. Amend J. P. (2014). Activity and phylogenetic diversity of sulfate-reducing microorganisms in low-temperature subsurface fluids with the upper oceanic crust. *Front. Microbiol.* 5:748 10.3389/fmicb.2014.00748 PMC4295021 25642212

[B35] Rouxel O. J. Ono S. Alt J. Rumble D. Ludden J. (2008). Sulfur isotope evidence for microbial sulfate reduction in altered oceanic basalts at ODP Site 801. *Earth Planetary Sci. Lett.* 268 110–123. 10.1016/j.epsl.2008.01.010

[B36] Ryan W. B. F. Carbotte S. M. Coplan J. O. O’hara S. Melkonian A. Arko R. (2009). Global multi-resolution topograpjy synthesis. *Geochem. Geophys. Geosyst.* 10:Q03014 10.1029/2008GC002332

[B37] Santelli C. M. Edgcomb V. P. Bach W. Edwards K. J. (2009). The diversity and abundance of bacteria inhabiting seafloor lavas positively correlate with rock alteration. *Environ. Microbiol.* 11 86–98. 10.1111/j.1462-2920.2008.01743.x 18783382

[B38] Santelli C. M. Orcutt B. N. Banning E. Bach W. Moyer C. L. Sogin M. L. (2008). Abundance and diversity of microbial life in ocean crust. *Nature* 453 653–656. 10.1038/nature06899 18509444

[B39] Smith D. C. Spivack A. J. Fisk M. R. Haveman S. A. Staudigel H. Party L. S. S. (2000). Methods for quantifying potential microbial contamination during deep ocean coring. *ODP Technical. Note* 28 10.2973/odp.tn.28.2000

[B40] Stevens T. O. McKinley J. P. (1995). Lithoautotrophic microbial ecosystems in deep basalt aquifers. *Science* 270 450–454. 10.1126/science.270.5235.450

[B41] Suzuki M. T. Béjà O. Taylor L. T. Delong E. F. (2001). Phylogenetic analysis of ribosomal RNA operons from uncultivated coastal marine bacterioplankton. *Environ. Microbiol.* 3 323–331. 10.1046/j.1462-2920.2001.00198.x 11422319

[B42] Sylvan J. B. Sia T. Y. Haddad A. G. Briscoe L. J. Toner B. M. Girguis P. R. (2013). Low temperature geomicrobiology follows host rock composition along a geochemical gradient in Lau Basin. *Front. Microbiol.* 4:61 10.3389/fmicb.2013.00061 PMC3608910 23543862

[B43] Takai K. Campbell B. J. Cary S. C. Suzuki M. Oida H. Nunoura T. (2005). Enzymatic and genetic characterization of carbon and energy metabolisms by deep-sea hydrothermal chemolithoautotrophic isolates of Epsilonproteobacteria. *Appl. Environ. Microbiol.* 71 7310–7320. 10.1128/AEM.71.11.7310-7320.2005 PMC1287660 16269773

[B44] Takai K. Horikoshi K. (2000). Rapid detection and quantification of members of the archaeal community by quantitative PCR using fluorogenic probes. *Appl. Environ. Microbiol.* 66 5066–5072. 10.1128/AEM.66.11.5066-5072.2000 PMC92420 11055964

[B45] Templeton A. S. Knowles E. J. Eldridge D. L. Arey B. W. Dohnalkova A. C. Webb S. M. (2009). A seafloor microbial biome hosted within incipient ferromanganese crusts. *Nat. Geosci.* 2 872–876. 10.1038/NGEO696

[B46] Toner B. M. Santelli C. M. Marcus M. A. Wirth R. Chan C. S. Mccollom T. M. (2009). Biogenic iron oxyhydroxide formation at mid-ocean ridge hydrothermal vents: juan de Fuca Ridge. *Geochim. Cosmochim. Acta* 73 388–403. 10.1016/j.gca.2008.09.035

[B47] Ver Eecke H. C. Butterfield D. A. Huber J. A. Lilley M. D. Olson E. J. Roe K. K. (2012). Hydrogen-limited growth of hyperthermophilic methanogens at deep-sea hydrothermal vents. *Proc. Natl. Acad. Sci. U.S.A.* 109 13674–13679. 10.1073/pnas.1206632109 PMC3427048 22869718

[B48] Wheat C. G. Jannasch H. W. Fisher A. T. Becker K. Sharkey J. Hulme S. (2010). Subseafloor seawater-basalt-microbe reactions: continuous sampling of borehole fluids in a ridge flank environment. *Geochem. Geophys. Geosyst.* 11:Q07011 10.1029/2010GC003057

[B49] Wheat C. G. Jannasch H. W. Kastner M. Plant J. N. Decarlo E. H. (2003). Seawater transport and reaction in upper oceanic basaltic basement: chemical data from continuous monitoring of sealed boreholes in a ridge flank environment. *Earth Planetary Sci. Lett.* 216 549–564. 10.1016/S0012-821X(03)00549-1

